# Novel Hydrogel Material as a Potential Embolic Agent in Embolization Treatments

**DOI:** 10.1038/srep32145

**Published:** 2016-08-26

**Authors:** Feng Zhou, Liming Chen, Qingzhu An, Liang Chen, Ying Wen, Fang Fang, Wei Zhu, Tao Yi

**Affiliations:** 1 Department of Neurosurgery, Huashan Hospital, Fudan University, Shanghai 200040, China; 2 Department of Chemistry and Collaborative Innovation Center of Chemistry for Energy Materials, Fudan University, Shanghai 200433, China

## Abstract

We report a novel graphene-oxide (GO) enhanced polymer hydrogel (GPH) as a promising embolic agent capable of treating cerebrovascular diseases and malignant tumors, using the trans-catheter arterial embolization (TAE) technique. Simply composed of GO and generation five poly(amidoamine) dendrimers (PAMAM-5), our rheology experiments reveal that GPH exhibits satisfactory mechanical strength, which resist the high pressures of blood flow. Subcutaneous experiments on Sprague-Dawley (SD) rats demonstrate the qualified biocompatibility of GPH. Finally, our *in vivo* experiments on New Zealand rabbits, which mix GPH with the X-ray absorbing contrast agent, Iohexol, reveal complete embolization of the artery. We also note that GPH shortens embolization time and exhibits low toxicity in follow-up experiments. Altogether, our study demonstrates that GPH has many advantages over the currently used embolic agents and has potential applications in clinical practice.

Cerebrovascular diseases and malignant tumors have become a global burden to human health and development, and are ranked within the top five leading causes for death in many countries, especially well-developed ones^1^,^2^,^3^. As a new and fast developing technique, trans-catheter arterial embolization (TAE) has emerged as one of the safest and most efficient methods in treating these broad diseases^4^,^5^,^6^. On one hand, TAE can help block the diseased or damaged arteries in brain, which, without timely treatment, can lead to mortality. In fact, TAE is an ideal or preferred therapy in cerebrovascular diseases such as arteriovenous malformation (AVM) and intracranial aneurysm^7^,^8^,^9^,^10^. On the other hand, injecting embolic agents into the feeding artery has been shown to inhibit tumor growth, by limiting the nutrition available at the tumor site. As such, embolization therapy has been widely adopted in different tumor treatments, successfully achieving safe and efficient results^11^,^12^,^13^,^14^.

Embolic agents greatly influence the result of treatment and are considered a key factor in developing the TAE technique. Currently, there are a variety of embolic agents available in clinics, divided into solid embolic materials and liquid embolic materials. However, none are regarded as ideal because of the associated side effects and complications such as infections^15^, spasms^16^, arterial rupture^17^, recanalization^18^ and even death^19^. What’s more, high recurrence and recanalization rates have encouraged surgeons to develop new materials for permanent embolization.

Solid embolic materials, such as gelatin sponges, microfibrillar collagen, surgical silk sutures, detachable balloons and coils are usually placed into target arteries. They can only be transported in large arteries and are unable to reach to the smaller branches; solid materials can only fulfill less than 30% of target arteries^20^, which causes high recurrence and recanalization rates and patients have to repeatedly receive treatment^21^,^22^.

In contrast, liquid embolic materials, such as cyanoacrylates, ethylene vinyl alcohol copolymer mixtures (EVAL), poly(vinyl acetate), cellulose acetate polymer, and poly(vinyl alcohol) (PVA), are usually dissolved in organic solvent and injected into target arteries^23^. Once the solution is injected into target arteries and the solvent is released into the blood, solid implants are formed by mechanisms including polymerization, precipitation and cross-linking through ionic or thermal process^24^. The only solution approved by the Food and Drug Administration (FDA), Onyx gel, contains ethylene vinyl alcohol dissolved in dimethyl-sulfoxide (DMSO) with Tantalum powder as the contrast agent for digital subtraction angiography (DSA) guidance. Though a stable gel forms after Onyx gel releases DMSO into the blood, the powerful solvent not only requires a specially designed catheter to resist dissolving, but also exhibits local and systemic cardiovascular toxicity^25^,^26^,^27^,^28^. This requires Onyx gel to be injected as slowly as 1.5 mL in 30 minutes to avoid DMSO delivery in blood flow and allow the polymer plug become stable enough to resist blood flow^29^. Besides, while Tantalum powder exhibits good X-ray absorbing ability to guide embolization, it can form artifacts that disturb the angiography.

We therefore conclude that an ideal permanent embolic agent should exhibit both mechanical and biochemical stability (mechanical stability would resist the blood flow and reduce the possibility of recanalization, and biochemical stability means non-biodegradable or irreducible because recurrence and recanalization means the failure of the treatments). Moreover, the agent should be absent of-or at least lower in–toxicity compared with existing materials, which require more biocompatible composition to avoid cardiovascular toxicity or strong immune reaction. Finally, the agent should be surgery-friendly to achieve good embolization results. Well-designed injectable embolic agents, if mixed with X-ray absorbing contrast agents, can easily meet all the requirements of TAE (such as efficient delivery to target arteries, fast setting process without adhesion to catheter, good visibility by DSA and 100% embolization results).

Taking all of these factors into account, we believe that an enhanced hydrogel may be the most promising choice as an ideal embolic agent, exhibiting the advantages of both solid and liquid embolic materials. Enhanced hydrogels are often described as solid embolic materials “dissolved” in H_2_O. Here, we define it as a hydrogel embolic material. Since the contrast agent, Iohexol, is also soluble in water, the hydrogel can be seen easily in DSA guided TAE, and is more biocompatible than liquid embolic materials dissolved in organic solvent. Moreover, with appropriate gel composition, hydrogels can also achieve good syringe ability and strong mechanical properties.

Based on our experiences of graphene oxide (GO), we propose that GO-enhanced hydrogels could be the ideal candidate for such an agent. Graphene and its derivatives are regarded as promising materials for important clinical applications in the fields of drug delivery, bio-sensing^30^, tissue engineering^31^ and diagnosis^32^,^33^. In particular, GO has attracted tremendous attention because of its outstanding electronic, thermal, optical and mechanical properties^34^. Subsequently, in addition to clay and cellulose carbon nanotubes, GO is now regarded as an effective additive to improve the mechanical performance of hydrogels^35^. Zhang, *et al*.^36^ and Liu, *et al*.^37^ have previously introduced GO into networks of polyacrylamide (PAM), demonstrating its contribution to improving the mechanical properties of the hydrogels.

In this work, we have designed a novel hydrogel embolic material, based on a GO-enhanced polymer hydrogel (GPH), which is composed of GO and generation five poly(amidoamine) dendrimers (PAMAM-5). Through rheology experiments and subcutaneous injection on Sprague-Dawley (SD) rats, we prove that the GPH possesses acceptable mechanical stability and good biocompatibility. The *in vivo* endovascular studies on New Zealand rabbits further confirm its potential use in TAE. Through our study, we demonstrate the feasibility of hydrogel embolic materials, and set the scene for further investigations into the practical applications of these hydrogel implants for use in clinic.

## Results and Discussion

### Formation and composites of GPH

**Figure 1:**
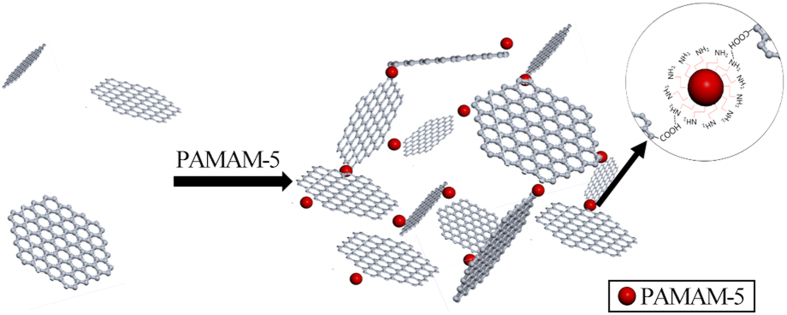
Schematic diagram showing the formation of GPH2.

GPH was chemically linked by carboxylate of GO and amine group of PAMAM-5 (Fig. 1). Similar composites have already been adopted to deliver and release drugs at low concentrations, with good syringe ability^38^. Here, we increased the concentration of GO to obtain considerable mechanical strength.

To find optimized composites of GPH with both mechanical stability and syringe ability, we prepared a series of samples (GPH1, 2, 3, 4, 5, 6 and 7) with different compositions of raw materials as shown in Table 1. Rheology experiments demonstrated that the influence of different GPH composites did not remarkably affect their mechanical properties.

**Figure 2:**
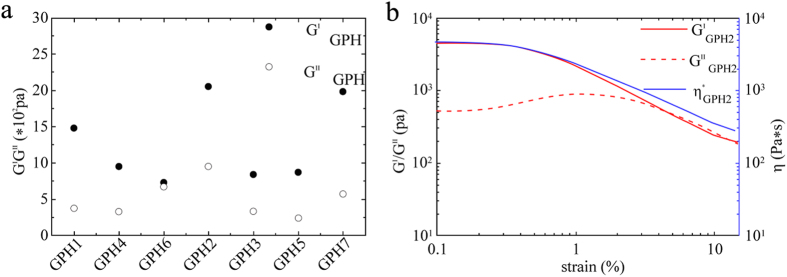
(**a**) The comparison of changes of the storage modulus and loss modulus (15 min after the addition of GDL) of GPH2 with other GPH samples. (**b**) Strain dependent storage modulus, loss modulus, and compressive modulus for GPH2.

GPH2 showed acceptable mechanical stability (storage and loss moduli were 2050 Pa and 952 Pa, respectively, shown in Table 1), and marginally better mechanical properties compared with most other composites of GPH (shown in Fig. 2a). Inappropriate NaOH/GDL ratio (GPH1, GPH3, GPH4, and GPH5 vs. GPH2) affected the mechanical strength by changing the pH of the gel. The increase of PAMAM-5 strengthened the network of the gel but affected the injectability. The storage and loss moduli of GPH2 both decreased and even inversed under some circumstances (shown in Fig. 2b) as the strain increased due to its structure. The proper mechanical strength gave GPH2 a better injectability even as it formed a hydrogel. The rheology experiments confirmed that GPH2 was the best composite choice for its considerable mechanical stability against shear force and gelation process. Therefore, we focused the rest of our investigations on GPH2.

### Characterization of GPH2

We conducted (X-ray photoelectron spectroscopy) XPS experiments to determine the chemical elements of the GPH2 hydrogel (shown in Figure S1a). The appearance of N in GPH2 (9.22%) demonstrated the existence of PAMAM-5. Raman spectroscopy revealed the ratio of sp^3^ hybridized C vs. sp^2^ hybridized C, which is represented as *I*_*D*_/*I*_*G*_. As shown in Figure S1b, the *I*_*D*_/*I*_*G*_ value of GPH2 (94.8%) was slightly higher than that of pure GO (92.6%), indicating the introduction of PAMAM-5 into GPH2 hydrogel.

The commonly used embolic agent, Onyx, not only requires DMSO as the solvent and Tantalum powder as the contrast agent for embolization, but it also begins embolization using the remaining ethylene vinyl alcohol (EVOH) when DMSO is released into the blood flow, thereby, small hydrogel fragments are left floating around for a little while^39^. In contrast, since GPH2 is a hydrogel with strong mechanical properties, it uses water as a more biocompatible solvent and the water-soluble contrast agent, Iohexol, as an easily metabolized contrast agent compared with Tantalum powder. Based on these findings, we next conducted animal experiments to determine the bio-toxicity of GPH2 and study its potential application in clinic.

### SD rat experiments

To detect the preliminary bio-toxicity of GPH2, we conducted subcutaneous injections on SD rats before DSA guided embolization *in vivo*. After injection, all of the rats maintained excellent reaction and action capacity. Hematological analysis revealed the general condition of the body (Figure S2), while histopathological analysis reflected the regional effect of the subcutaneous injections (Figure S3). The results showed that GPH has satisfactory biocompatibility and insignificant toxicity.

One day after injection, there was no acute immune reaction at the injection site. Only few inflammatory cells appeared around the hydrogel, most likely related to the mechanical compression (shown in Figure S3a). The red blood cell count (8.48 ± 0.66 × 10^12^/L), white blood cell count (4.96 ± 0.85 × 10^9^/L) and hemoglobin count (157.00 ± 5.00 g/L) did not differ significantly with the control group. The platelet count (812.0 ± 64.09 × 10^9^/L) was slightly lower than the control group (1010.2 ± 98.47 × 10^9^/L), but within the 95% confidence interval. We examined the data using the reference value reported by Petterino in 2006^40^.

An acute immune reaction peaked on the fifth day after the injection. In the HE staining slides, GO was scarlet in color and exhibited a clear shape, which was easily distinguished from the tissues around the hydrogel. The foreign body reaction was active since the macrophages, inflammatory cells and fibroblasts can be easily found around the injection site (shown in Figure S3c). Several immune cells infiltrated into the hydrogel. However, the cell count in the hematological analysis (erythrocyte: 8.34 ± 0.41 × 10^12^/L, leukocyte: 5.34 ± 1.42 × 10^9^/L, platelet: 943.67 ± 30.62 × 10^9^/L, hemoglobin: 149.0 ± 3.61 g/L) did not differ significantly from one another. These results suggest no significant infections or inflammation of the body. Therefore, the inflammatory response was partial and limited.

**Figure 3:**
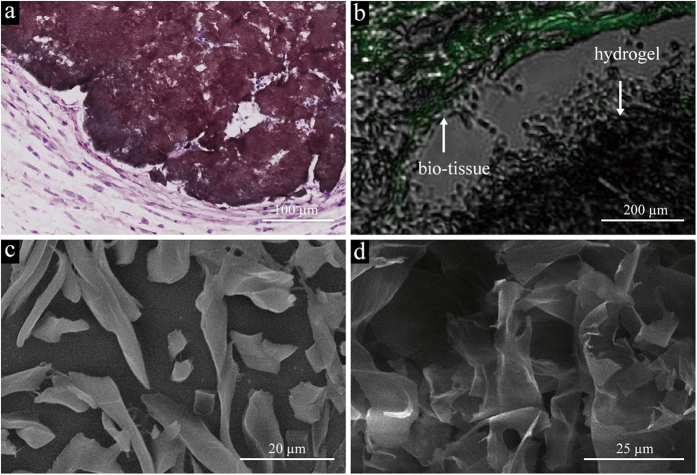
(**a**) HE staining microscopy images of GPH2 four weeks after subcutaneous injection. A small amount of inflammatory response is shown around the graphene oxide. (**b**) CLSM images of GPH2 four weeks after subcutaneous injection. The inset shows the clear border of the tissues and GPH2. (**c**) SEM images of GPH2 compounds *in vivo* after subcutaneous injection for four weeks. (**d**) SEM image of pure GPH2.

Four weeks after injection, the hematological analysis items (erythrocyte: 8.21 ± 0.14 × 10^12^/L, leukocyte: 10.82 ± 3.68 × 10^9^/L, platelet: 966.67 ± 159.36 × 10^9^/L, hemoglobin: 154.00 ± 6.08 g/L) did not differ significantly with the control group and reference value. The injected materials became granulomas. We found obvious fibrosis around the injection site (Fig. 3a) and an active foreign body giant cell response. However, the inflammatory response was still limited.

We applied (Confocal Laser Scanning Microscope) CLSM to prove the existence of GO, which can be seen as black in both bright and fluorescent fields (shown in Fig. 3b), while the tissues were green in fluorescent field.

SEM images provided a more detailed structure of the subcutaneously injected hydrogels. The morphology of pure GPH2 hydrogel consisted of disordered sheets of tens of micrometers (Fig. 3d)^41^,^42^,^43^,^44^ assembled by GO sheets adhered by PAMAM-5. The existence of bio-liquids means that the injected hydrogels were wrapped within the components inside, which potentially caused the different morphology (Fig. 3c).

From the subcutaneous injection and hematological analysis, we can conclude that the main immune reaction was a foreign body response, which peaked on the fifth day after injection, and no infection, necrosis and poisoning were found. Moreover, composites of GPH2 were non-biodegradable and GPH2 became granulomas forming a significant boundary with tissues, which meets the requirements for permanent embolization.

### DSA guided embolization

**Figure 4:**
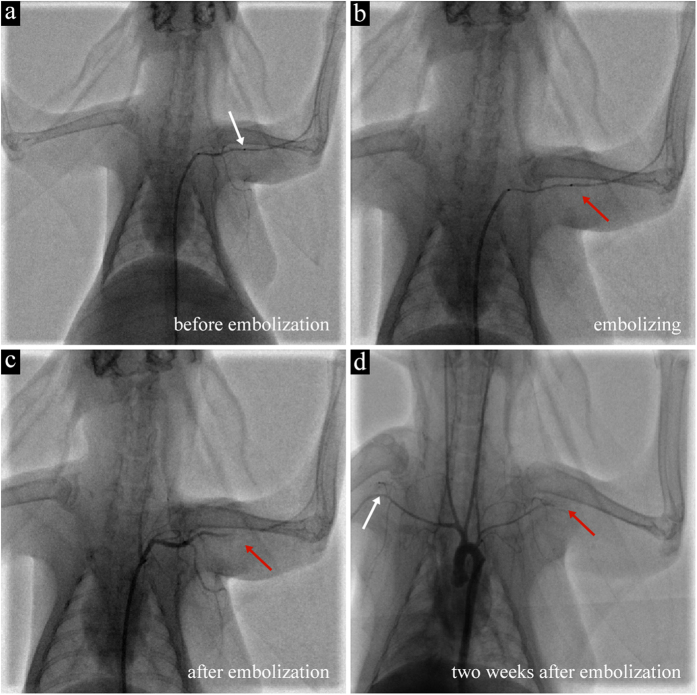
(**a**–**d**) Screen capture of DSA embolization. (**a**) Before embolization, the contrast agent can pass the subclavian artery. (**b**) GPH was injected smoothly into the target artery. The red arrow points to the micro catheter’s mouth. (**c**) After embolization, the contrast agent cannot infuse the distal segment of the subclavian artery. It turns back at the embolization site. (**d**) Two weeks later, the contrast agent still stops at the embolization site (the red arrow indicates the end of the artery stops at the collarbone and no artery can be seen under the collarbone). The contrast agent can pass the right subclavian artery smoothly (white arrow; under the collarbone).

Based on the results of the SD rat experiments, we next replicated the clinical embolization treatment in four New Zealand rabbits (Video 1). Before embolization, the contrast agent was passed through the subclavian artery (Fig. 4a). When the GPH2 was injected into the subclavian artery, it was clearly distinguished from the body tissue, due to efficient mixing of contrast agent with GPH2. The hydrogel passed through the catheter smoothly without the affection of blood flow, blocking the artery within few minutes (Fig. 4b). After embolization, the contrast agent was unable to pass the subclavian artery, which indicated that the target vascular was completely blocked (Fig. 4c). A video is provided in the supporting materials showing the injecting process with Iohexol, and screen shots are shown in Fig. 4a–c.

After embolization, four rabbits were kept for two weeks for follow-up investigations. None of the animals died or had serious complications during the observation period.

We identified three main criteria to determine the treatment results:

#### Clinical manifestations

We observed significant clinical features during the two-week period. Embolization of the subclavian artery demonstrated obvious symptoms to the rabbit limb. The body temperature was lower than the other healthy limb and the skin became darker, due to the lack of blood (shown in Figure S4a,b). However, physical activity of the limb was not affected.

#### Imaging findings

The review of DSA found that the subclavian artery was totally blocked without recanalization (shown in Fig. 4d). The contrast agent mixed with GPH had already metabolized and no artifacts in the angiography were observed after two weeks (shown in Video 1).

#### Pathological examinations

**Figure 5:**
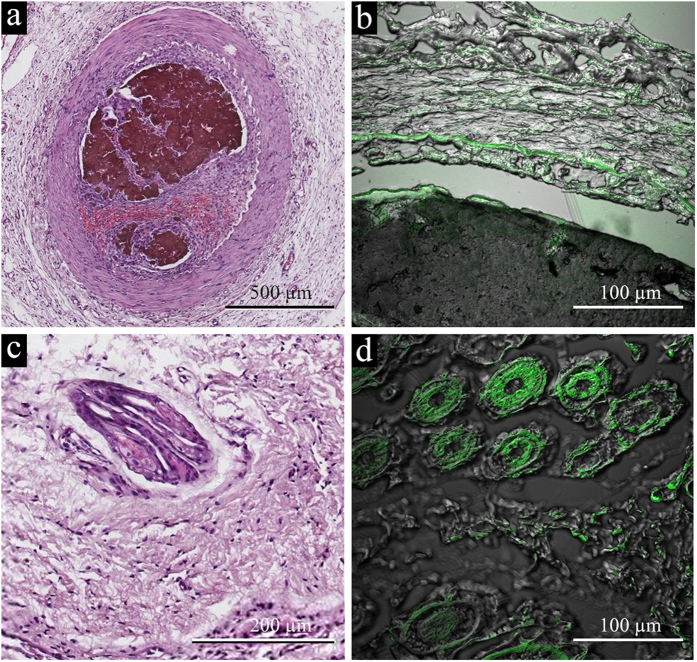
(**a**) HE staining image of GPH2 after DSA embolization. The artery is completely blocked. (**b**) CLSM image of GPH2 after embolization. (**c**) HE staining microscopy image of left paw tissue after embolization. No inflammation and fragments of GPH can be seen. (**d**) CLSM image of left paw tissue after DSA embolization.

Our HE staining and CLSM images of the subclavian artery showed a complete blockage of the vascular by the GO hydrogel (Fig. 5a,b). As with our HE staining experiments in SD rats, we observed the hydrogel to be scarlet in color. We noted that large amounts of inflammatory cells and macrophages formed the granuloma in the artery and fibroblasts were active around the vascular. Therefore, recanalization of the artery was unable to occur.

Through these three criteria, we can determine that the GPH was efficient in embolization and the treatment exhibited satisfying results.

### Limitations and special concerns

Before the GPH is applied in further studies or for practical use, we recommend acknowledging the following concerns.

#### Toxicity

The potential toxicity of GO in GPH is under great academic consideration. Some studies have reported that GO is safe at low concentration^45^,^46^,^47^, while others have noted that GO exhibits dose-dependent toxicity in animals and cells. In a chronic toxicity study, mice (28–30 g) were injected with different doses of soluble GO. GO under low dose (0.1 mg) and middle dose (0.25 mg) did not exhibit obvious toxicity to mice, but under high dose (0.4 mg) exhibited chronic toxicity in organs included the lungs, liver and kidney^48^. The safe dose was reported as 0.25 mg per 28 g (8.93 mg/kg). In our study, each New Zealand rabbit was injected with no more than 1 mL GPH, which was more than enough to block the target artery. The weight of the rabbits ranged from 3.0 kg to 3.5 kg. Therefore, the dose was 3 mg/kg, which is lower than the reported safe dose. The hydrogel is localized to the specific area and does not spread through as with a solution. We designed the following experiments to investigate the GO and its potential pathological influence in the related tissues and organs.

##### Blood circulation

In order to determine whether fragments of hydrogel were floating in the blood, which, due to being composed of GO, might be harmful to different organs, we collected serum samples of the rabbit before, right after, and two weeks after the injection and studied them by Raman experiments. We did not observe any GO signals in the serum samples (Figure S5), which indicates that no hydrogel fragments were found floating in the blood.

##### Target tissues

We also took tissue samples from the end of the embolization limb (the paw, muscle and skin) for histopathological detection and did not report any significant pathological changes (Fig. 5c). The corresponding CLSM images are shown in Fig. 5d, which also confirm the absence of GO.

##### Organs with rich capillaries

**Figure 6:**
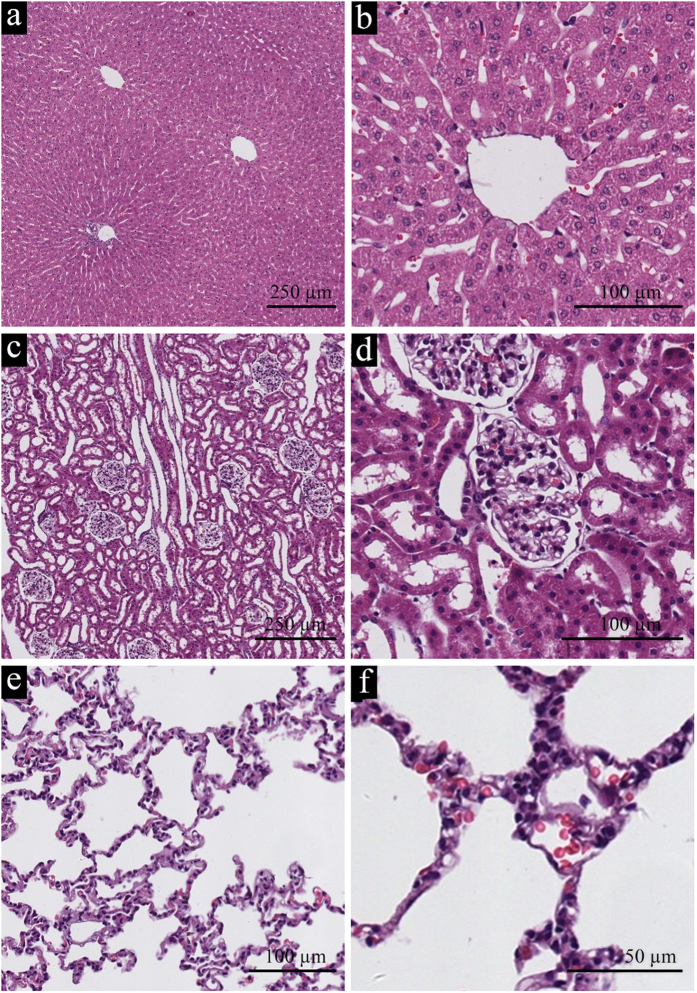
(**a**,**b**) HE staining images of liver tissue. (**c**,**d**) HE staining images of kidney tissue. (**e**,**f**) HE staining images of lung tissue.

Important organ samples were also sent for histopathological examination, which revealed no significant pathological changes. The hepatocyte did not exhibit any serious cell degeneration or necrosis. The structure of the hepatic cord could be clearly observed in the slides (Fig. 6a,b). Similarly, no pathological changes were found both in renal cortex and medulla tissues, while the structure of the renal corpuscle and glomerulus did not exhibit any specific changes (Fig. 6c,d). Only a few erythrocytes were found in the tubules. We did not observe any inflammation or tumors in the lungs and the structure of the respiratory bronchiole and pulmonary alveoli did not differ significantly from normal tissue (Fig. 6e,f).

Through these experiments, we primarily determined the existence and pathological changes of GO in the investigated organs and tissues. However, we recommend including a larger selection of tissues and organs in future studies. To comprehensively study the toxicity of GO as a permanent implanted agent in the body, we plan to introduce further studies on the molecular mechanism and the materials-cell effect. Recent toxicity study showed that nanoscale GO had a dose-dependent effects on reproduction capability of mammals^49^. Size-dependent genotoxicity of graphene nanoplatelets was also confirmed in human stem cells^50^. GO not only acts as a permanent embolic agent but also takes part in metabolism in the body. Though no significant pathological changes were found in the slides, a longer follow-up time and investigation on the molecular biological mechanism should be included in further investigations.

#### Material-cell interaction

Implanted materials affect the micro-environment of neighboring tissues. We showed that interactions between cells and the GPH demonstrate permanent embolism once the embolic agents are implanted. Particle size and the composite of GPH also influence the contacted cells, while the chemical state of GO of the composite can also change after interaction by active cells and their media^51^.

In our study, we investigated the hydrophilicity and lipophilicity of the GPH to determine the surface of the contact. Through contact angle measurements, we found that the surface of dry gel was amphipathic (Figure S6), which was related to the composite (GO and PAMAM-5) and micro-structure. Constant exchanges between the cells and materials may be conducted by the activities like endocytosis, exocytosis and immune reaction. Furthermore, like the onyx gel, the irregular shapes of the embolic agent may result in incomplete embolization right after the surgery. With the material-cell interaction, the immune response and the organized thrombus would fill in the blanks in the following postoperative days. HE slides and pathological examinations confirmed that the active fibrosis and thrombus blocked the artery completely (Figs 5a and S7). The embolic agent acted not only as an embolic agent but also an irritant to stimulate the body reaction. We are now introducing immunohistochemistry detection to study the relationship between the material and cells in the micro-environment.

Besides, vessel and tumor types can limit the efficiency and application of embolic agents. Different treatment results may be achieved in special anatomical structures like blood brain barrier (BBB), collateral circulation and artery plexus. Embolization in animal disease models should be conducted to confirm GPH treatment results in varied hemodynamic environment.

Like onyx gel, GPH also required premixing before the surgeon locates the micro catheter at the appropriate site. We shortened the premix time from 20 minutes into 2 minutes and saved preparation steps to the micro catheter necessary in onyx use. The GPH can be injected through the common micro catheter without the help of other assistant equipment, such as a balloon. Furthermore, the GPH can be injected at any speed, whereas onyx is required to be injected at less than 0.3 ml per minute. However, the surgeon was required to inject the GPH at a certain time before the hydrogel clogs the micro-catheter, which is the main technique limitation of GPH injection.

## Conclusion

In conclusion, our study reveals GPH2 as a potential clinical embolic agent in TAE treatments. Our chemical characterization and biological experiments prove that GPH2 is biocompatible and exhibits several advantages over currently used embolic agents. Firstly, unlike Onyx, GPH2 does not release any organic solvent to the blood, which reduces potential complications. Secondly, GPH2 blocks the artery in a short time without solid implant formation *in vivo*. Thirdly, similar to liquid embolic materials, our hydrogel embolic agent fills nearly 100% of the target vascular, and therefore has lower possibility for recanalization. Fourthly, unlike normal liquid embolic agents, the mechanical properties of GPH2 guarantee no crush or transfer after injection. Lastly, GPH2 absorbs X-ray simply by adding water-soluble contrast agents that are quickly metabolized and leave no trace artifacts that may hinder future imaging examinations.

Despite the excellent performance of GPH2 as an embolic agent for potential applications in DSA guided TAE treatments, the functions and safety of the hydrogel require further improvements before they are introduced into clinical applications. In order to fulfill this goal, our research group now seeks to conduct further studies and find more potential candidates for hydrogel embolic agents.

## Methods

### Preparation of hydrogels

GO was prepared according to a modified Hummers method from natural graphite powder, as previously reported by our group^52^. The GO was dialyzed to remove any unreacted impurities. We obtained GO solution at various concentrations by sonicating GO in water.

A series of GPH hydrogels with different composites, as shown in Table 1, were prepared using similar approaches. Herein, we describe the preparation of GPH2 in detail as an example. To prepare GPH2, we added sodium hydroxide (NaOH, 1 mol/L, 400 μL) and PAMAM-5 (250 mg/mL, 200 μL) solution to GO (9 mg/mL, 2.5 mL) solution successively, followed by sonication in iced water to achieve uniform distribution of each addition. We then added newly prepared glucono-delta-lactone (GDL, 200 mg/mL, 200 μL) solution to the above mixture, which was then stirred. After incubation for a few minutes, the GPH2 hydrogel was formed. Here GDL served as a re-activator for carboxyl groups on GO passivized by NaOH, since it can release H^+^ slowly during its hydrolytic process.

### Instruments and characterizations

We performed XPS experiments on a RBD upgraded PHI-5000C ESCA system (Perkin Elmer) with Mg Kα radiation (*hν* = 1253.6 eV) or Al Kα radiation (*hν* = 1486.6 eV). Binding energies were calibrated using the containment carbon (C1s = 284.6 eV). We then studied carbon, nitrogen and oxygen elements inside the hydrogels. Before the experiments, we freeze-dried samples of GO and GPH2 under vacuum overnight.

We measured Raman spectra on an XploRa laser Raman spectrometer (manufactured by HORIBA Jobin Yvon, France) at room temperature with attenuation of excitation light source to prevent decomposition of polymer components inside the hydrogels. We freeze-dried the samples under vacuum overnight and calculated *I*_*D*_/*I*_*G*_ with the spectra at one time magnification.

We measured rheology experiments of GPH of different composites (Table 1) on an ARES Rheometer (manufactured by TA, USA). We began measuring GPH hydrogels at the addition of GDL. The strain and the frequency were set as 1% and 1 rad/s, respectively. Complex viscosity (η^*^) was calculated by Equation 1:


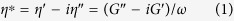


Scanning electron microscope (SEM) images of GPH2 were shot on a VEGA TS 5136 MM (manufactured by TESCAN, Czech Republic). We freeze-dried samples under vacuum overnight and sprayed them with gold.

Confocal laser scanning microscope (CLSM) imaging was performed with an OLYMPUS FV1000 laser-scanning microscope and a 60× oil-immersion objective lens. We excited prepared samples at 405 nm to see the background fluorescence and at 458 nm to see the bright field.

Surface wettability of the GPH was characterized by static water contact angle measurement. The image of the droplet on the GPH was visualized through the image analyzer (OCA40, Dataphysics, German) and the angle between the water droplet and the surface was measured. The GPH samples were dried and stored in a desiccator at ambient temperature before the measurements. An ultrapure water droplet of 6 μL was placed on the film and the recording was taken 30 s after the application of the droplet. Three measurements were made at different samples and an average value was calculated by statistical method. We also replaced the water with peanut oil to determine the angle between the droplet and the surface.

### SD rat experiments

All animal experiments were performed according to procedures approved by Fudan University Committee on Animal Care and Use. To preliminarily detect the bio-toxicity of GPH2, we conducted subcutaneous injections on SD rats before TAE *in vivo*.

Adult female SD rats weighing from 250 g to 300 g were divided into four groups, distinguished as the acute group (one day after injection), sub-acute group (five days after injection), chronic group (four weeks after injection) and the control group. Each group consisted of three rats. The subcutaneous injections of hydrogels were conducted at a dose of 0.3 mL hydrogel per 200 g weight of rat with 1 mL disposable syringe (produced by Kindly Enterprise Development Group Co. Ltd, Shanghai, China). Considering the sol-gel transition process, we injected with GPH2 immediately after the addition of GDL, since it formed a gel quickly.

After the observation ended, we collected 1 mL blood from the inferior vena cava after anesthetizing and dissecting the rats. The blood was immediately placed into the anticoagulant tube (with 3.6 mg K_2_ EDTA) and then analyzed using an auto blood analyzer. We then analyzed erythrocyte (red blood cells, RBC), leukocyte (white blood cells, WBC), platelets (PLT) and hemoglobin (HGB) to evaluate the general condition of the rats.

We also collected tissue around the injected compounds after the rats were killed by cervical dislocation and placed them into formalin solution for three days. Pathological analysis was based on hematoxylin-eosin (HE) staining, CLSM and SEM of the tissue around injected compounds. Briefly, the samples were embedded in paraffin after they were dehydrated using ethanol and xylene. We cut sample slices with a thickness of 3.5 μm and prepared them for HE staining (stuck to glass slide), CLSM (stuck to class bottom cell culture dish, ϕ = 20 mm, produced by NEST Biotechnology Co. Ltd) and SEM (stuck to mica plate, and freeze-dried overnight).

### DSA guided embolization

The *in vivo* tests were conducted based on endovascular techniques. We performed embolization guided by X-ray. After anesthetizing the New Zealand rabbit by sodium pentobarbital (30 mg per kilogram weight), the micro catheter arrived at the target vascular from the femoral artery. The GPH2 hydrogel was injected rapidly within five minutes after the addition of GDL, while a clinical radiopaque diagnostic agent replaced three fifth of the H_2_O in hydrogel. We performed angiographic analysis to evaluate subclavian artery blood flow immediately after embolization. The procedure was performed via left femoral artery approach.

We observed four rabbits for two weeks after embolization. All animals were followed up with angiography 14 days after embolization. We conducted Raman experiments of serum to determine whether GO remained in the blood. We took tissue samples from the left subclavian artery, connective tissues around the embolization artery, the end of the limb (paw, skin and muscle) and important organs (liver, kidney and lungs). We sent all tissues for pathological examination.

### Ethical approval

All animal experiments were performed according to procedures approved by Fudan University Committee on Animal Care and Use.

## Additional Information

**How to cite this article**: Zhou, F. *et al*. Novel Hydrogel Material as a Potential Embolic Agent in Embolization Treatments. *Sci. Rep.* **6**, 32145; doi: 10.1038/srep32145 (2016).

## Supplementary Material

Supplementary Video

Supplementary Information

## Figures and Tables

**Table 1:** Different composites of GPH.

Composite^a^	GO (mL)	NaOH (μL)	PAMAM-5 (μL)	GDL (μL)	*G*′^b^ (×10^2^ Pa)	*G*″^b^ (×10^2^ Pa)
GPH1	2.5	400	200	100	14.8	3.75
GPH2	2.5	400	200	200	20.5	9.52
GPH3	2.5	400	200	300	8.4	3.35
GPH4	2.5	200	200	200	9.5	3.31
GPH5	2.5	600	200	200	8.7	2.39
GPH6	2.5	400	100	200	7.3	6.70
GPH7	2.5	400	300	200	19.8	5.72

^a^Concentrations of the composites: GO, 9 mg/mL; NaOH, 1 mol/L; PAMAM-5, 250 mg/mL; GDL, 200 mg/mL. ^b^The storage modulus (*G*′) and loss modulus (*G*″) of GPS with different composites listed here were measured 15 min after the addition of GDL.
