# Supplementary material for: Novel Hydrogel Material as a Potential Embolic Agent in Embolization Treatments
Source: Sci Rep. 2016 Aug 26;6:32145. doi: 10.1038/srep32145 (PMC4999878; doi:10.1038/srep32145)
Supplement: Supplementary Information [file srep32145-s2.doc]

**Supporting Information**

Novel Hydrogel Material as a Potential Embolic Agent in Embolization Treatments

*Feng Zhou, Liming Chen, Qingzhu An, Liang Chen, Ying Wen, Fang Fang, Wei Zhu*, Tao Yi**


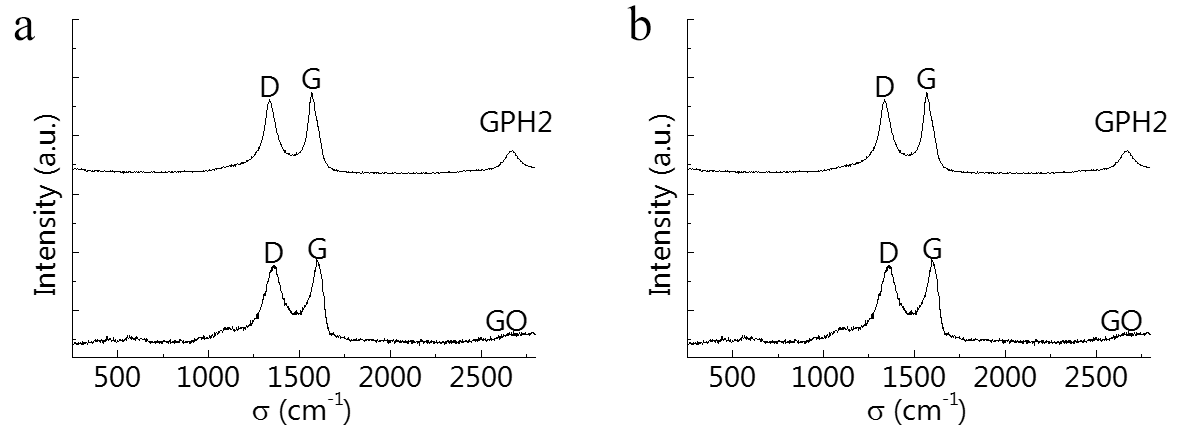


**Figure S1.** (A) X-ray photoelectron spectroscopy of GO and GPH2. (B) Raman spectroscopy of GO and GPH2.


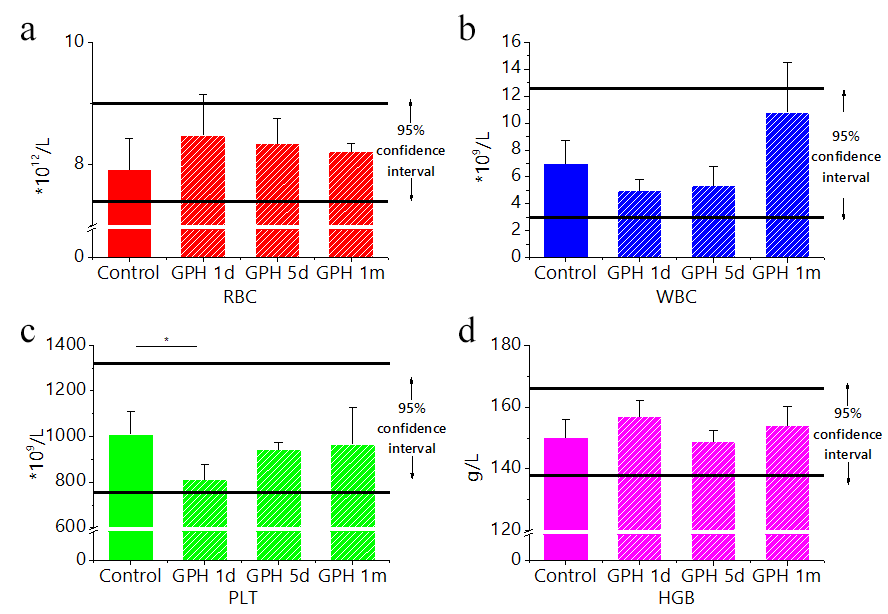


**Figure S2.** Hematological analysis. The 95% confidence interval can be seen between the two parallel lines (mean ± 1.96SD). The cell count of erythrocyte (A) and leukocyte (B) had no significant difference between the control group and the injection group. The platelet count (C) decreased after the hydrogel had been injected for one day (p=0.022), but it is still in the 95% confidence interval. In addition, no significant difference was found after injected for five days and four weeks. There is no significant difference of the hemoglobin (D) levels in all groups.


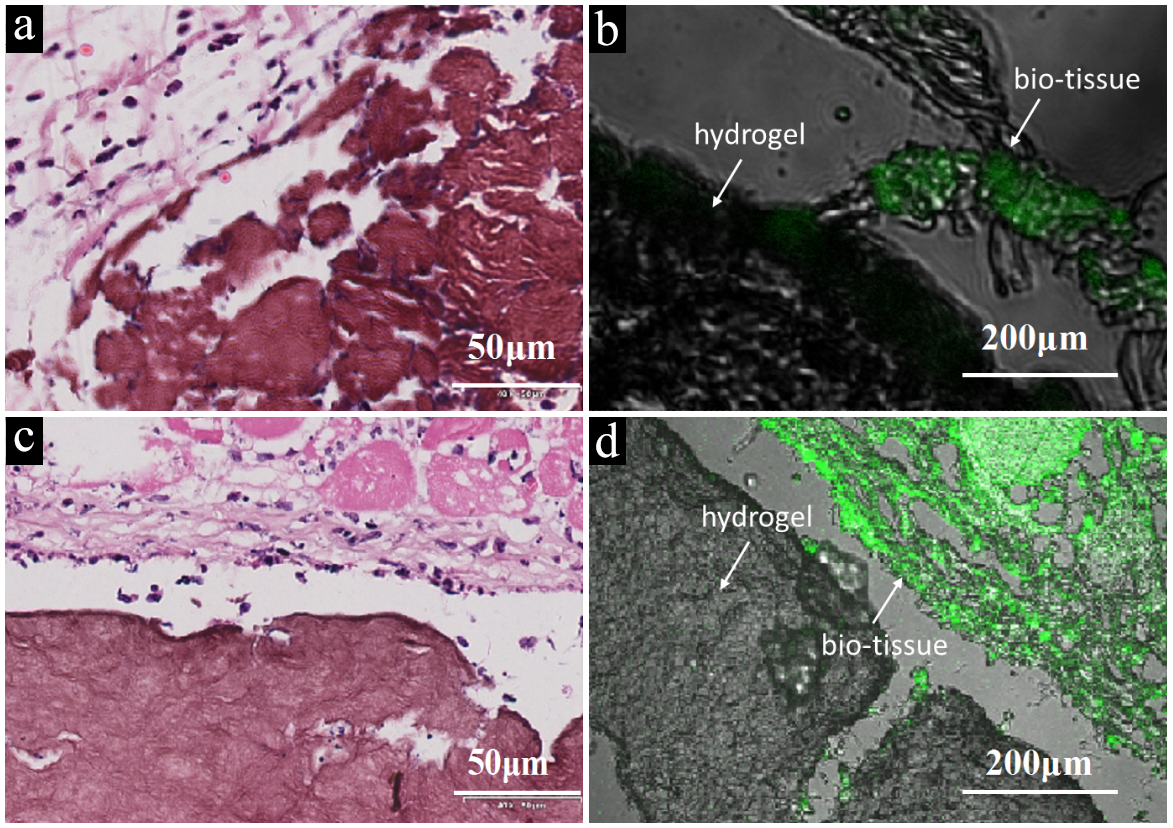


**Figure S3.** The pathological analysis of subcutaneous injection. HE staining microscopy images of tissues around injected compounds on the first day after injection of GPH2 (A), and on the fifth day after injection of GPH2 (C); corresponding CLSM images of the tissues around the injected compounds are shown to the left (B and D), showing clear borders between the tissue and GPH2.


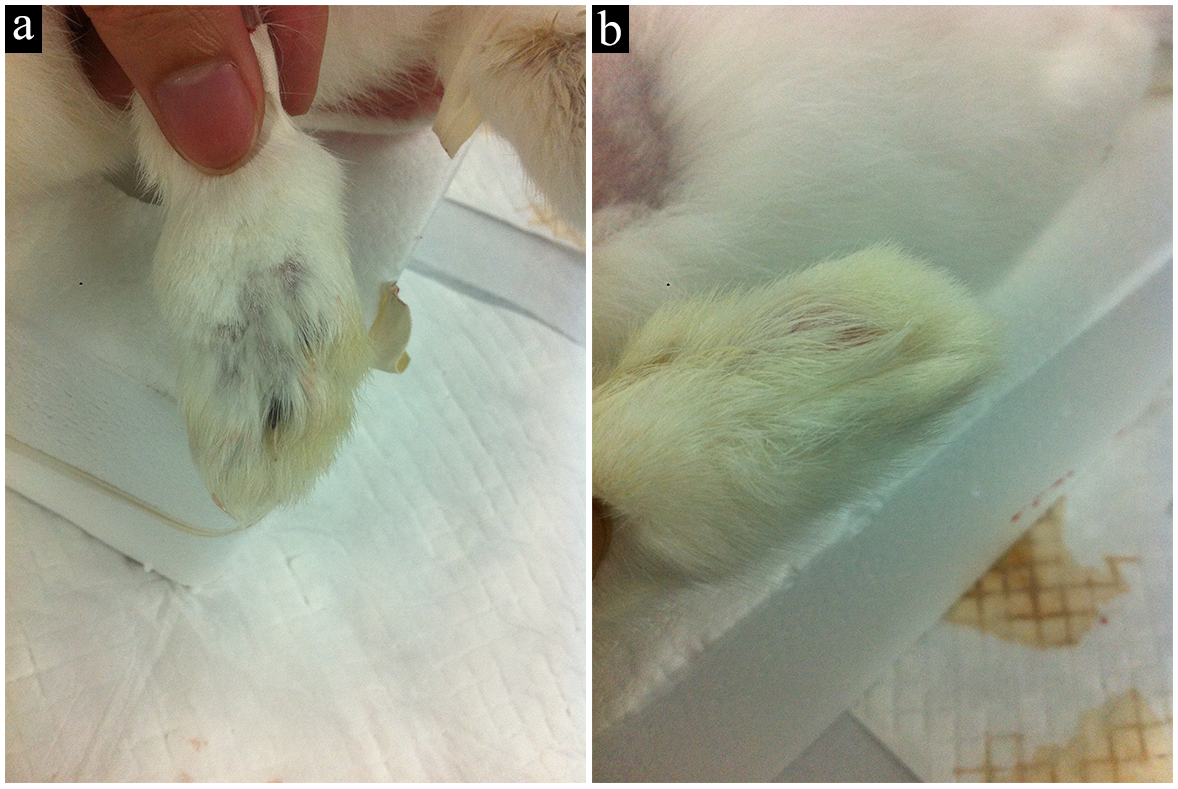


**Figure S4.** The skin of the blocked limb (A) and healthy limb (B). Due to the lack of blood, the blocked limb become black. The healthy limb also has a higher body temperature than the blocked one.


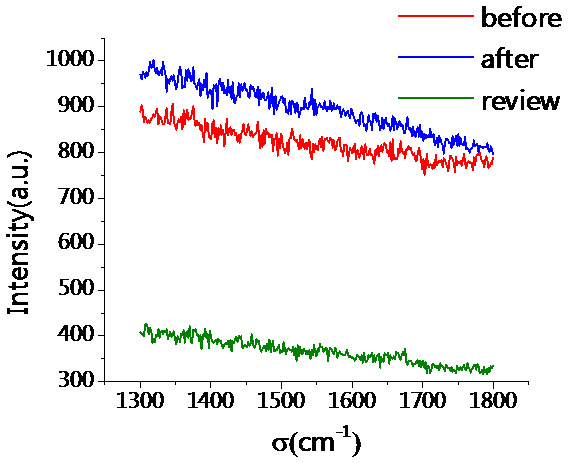


**Figure S5.** Raman spectroscopy of serum samples before embolization (red), a few minutes after embolization (blue) and two weeks after embolization (green).

**Figure S6.** Surface wettability of the GPH was characterized by static water contact angle measurements. (A) The angle between the water droplet and the surface is 32.9 ± 2.3°. (B) The angle between the oil droplet and the surface is 32.3 ± 4.3°. The results show that the surface of the dry gel is amphipathic.


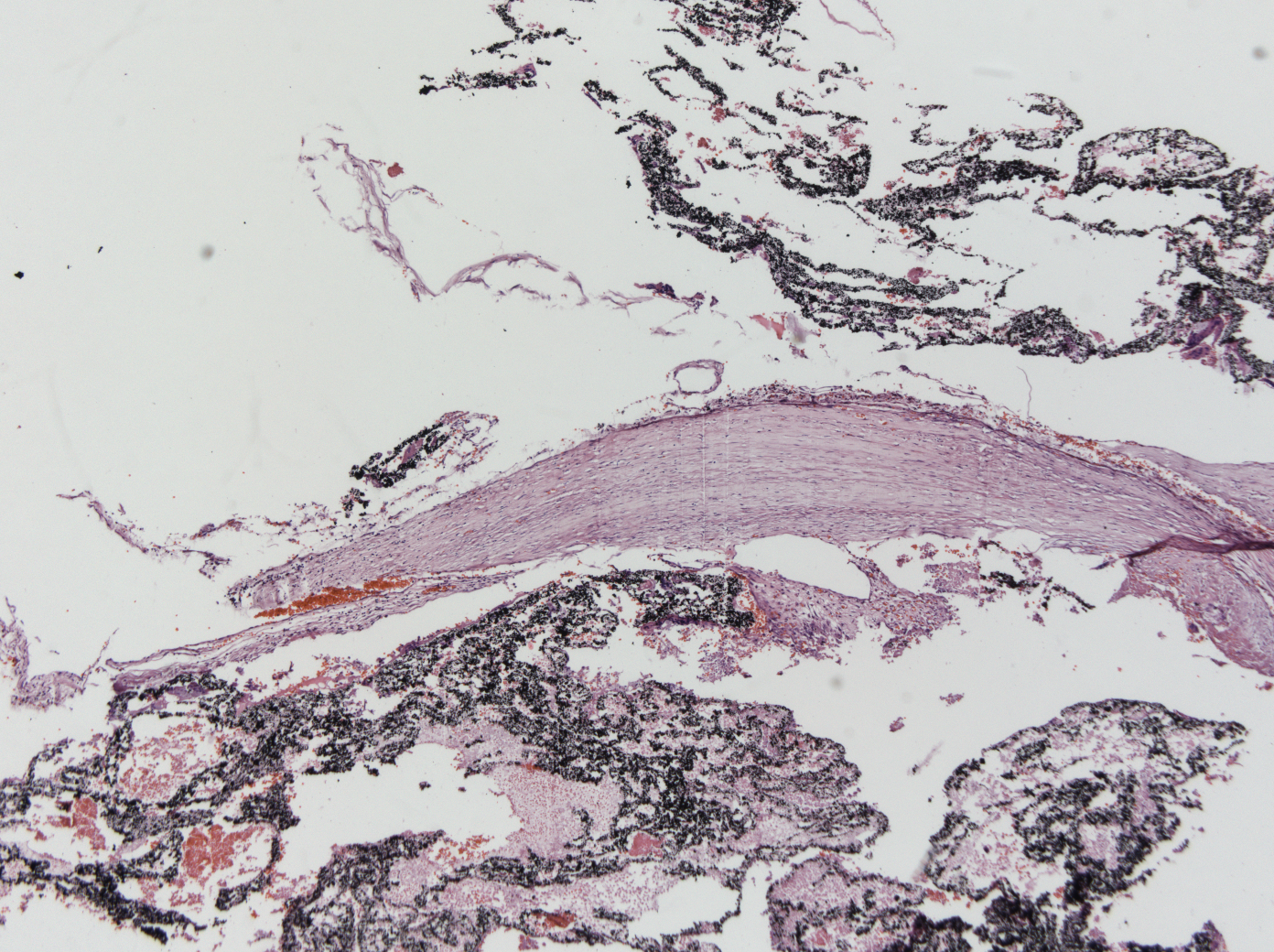


**Figure S7**. This figure shows the resection specimens from an AVM patient. The patient was treated with onxy embolization three months before accepting craniotomy. The HE slides (×40) show that the immune response and organized thrombus surround the arteries.

**Supplementary video legends**

This video showed the embolization process in the treatment. Before embolization, the contrast agent was passed through the subclavian artery. When the GPH2 was injected into the subclavian artery, it was clearly distinguished from the body tissue, due to efficient mixing of contrast agent with GPH2. The hydrogel passed through the catheter smoothly without the affection of blood flow, blocking the artery within few minutes. After embolization, the contrast agent was unable to pass the subclavian artery, which indicated that the target vascular was completely blocked. Two weeks later, the review of DSA found that the subclavian artery was totally blocked without recanalization. The contrast agent mixed with GPH had already metabolized and no artifacts in the angiography were observed.
